# Gravity-Based vs. Pump-Assisted Irrigation in Unilateral Biportal Endoscopic Decompression for Lumbar Spinal Stenosis: Effects on Operative Efficiency, Clinical Outcomes, and Complications

**DOI:** 10.3390/medicina62091640

**Published:** 2026-08-27

**Authors:** Uğur Özdemir, Abdülhalim Akar, Muhammed Fatih Serttaş, Ali Murat Başak, Tunahan Aka

**Affiliations:** 1 Department of Orthopaedics and Traumatology, Faculty of Medicine, Bandırma Onyedi Eylül University, 10200 Balıkesir, Türkiye; ahalimakar@gmail.com; 2 Department of Orthopaedics and Traumatology, Pendik Training and Research Hospital, Marmara University, 34899 İstanbul, Türkiye; mfserttas@gmail.com; 3 Department of Orthopedics and Traumatology, Gülhane Training and Research Hospital, 06010 Ankara, Türkiye; muratalibasak@gmail.com; 4 Department of Orthopaedics and Traumatology, Bandırma Training and Research Hospital, 10250 Balıkesir, Türkiye; tunahanaka034@gmail.com

**Keywords:** lumbar spinal stenosis, unilateral biportal endoscopy, irrigation, pump-assisted irrigation, operative time, complications

## Abstract

*Background and Objectives*: Unilateral biportal endoscopic (UBE) decompression has become an increasingly popular minimally invasive technique for the treatment of lumbar spinal stenosis. Although irrigation management is a critical component of this procedure that may influence operative efficiency and irrigation-related complications, comparative evidence regarding irrigation systems remains limited. Therefore, this study aimed to compare gravity-based and pump-assisted irrigation systems in patients undergoing UBE decompression for lumbar spinal stenosis with respect to clinical and functional outcomes, operative efficiency, and complication rates. *Materials and Methods*: This retrospective study included 107 patients who underwent single-level UBE decompression for lumbar spinal stenosis between 1 June 2023 and 1 January 2026. Patients were allocated to either the gravity-based (*n* = 51) or pump-assisted (*n* = 56) irrigation group according to the availability of the pump-assisted irrigation device at the time of surgery. Pain and functional outcomes were evaluated using the Numeric Rating Scale (NRS) and Oswestry Disability Index (ODI). Operative time, bleeding control time, and perioperative complications were compared between the groups. A mixed-design ANCOVA was used to analyze the changes in clinical outcomes over time. Multivariable linear and logistic regression analyses were performed to identify the independent factors associated with operative time and complications. *Results*: Significant improvements in the NRS and ODI scores were observed in both groups during follow-up (time effect, *p* < 0.001 for both), with no significant differences between the irrigation methods (group effect, *p* > 0.05). Operative and bleeding control times were significantly shorter in the pump-assisted irrigation group (both *p* < 0.001). Multivariable linear regression analysis demonstrated that pump-assisted irrigation independently reduced operative time by approximately 8 min (*p* < 0.001), whereas bilateral decompression independently increased operative time by approximately 24.5 min (*p* < 0.001). Although the overall complication rate was lower in the pump-assisted irrigation group (12.5% vs. 27.5%), the difference was not statistically significant (*p* = 0.052). Multivariable logistic regression analysis also demonstrated a trend toward a lower risk of complications with pump-assisted irrigation (OR = 0.408, *p* = 0.096). *Conclusions*: Pump-assisted irrigation improved operative efficiency by reducing operative and bleeding control times while providing clinical and functional outcomes comparable to those of gravity-based irrigations. Although the reduction in complication rates did not reach statistical significance, pump-assisted irrigation may represent an effective irrigation strategy that improves operative efficiency and may contribute to surgical safety in UBE decompression surgery.

## 1. Introduction

Lumbar spinal stenosis (LSS) is one of the most prevalent degenerative spinal disorders, affecting approximately 103 million individuals worldwide [1]. Its prevalence increases substantially with advancing age, and LSS is the leading indication for spinal surgery in patients older than 65 years [2]. As the global elderly population continues to grow, the demand for surgical treatment of LSS is expected to increase markedly in the coming decades [2]. Although the primary goal of surgical treatment is to achieve adequate neural decompression by addressing the underlying pathology, patient-centered outcomes, such as shorter hospital stays and faster functional recovery, have become increasingly important in determining the optimal surgical approach.

Accordingly, unilateral biportal endoscopic (UBE) surgery has become one of the most widely preferred minimally invasive approaches, particularly for treating LSS [3]. Compared with conventional open spine surgery, this technique preserves the surrounding anatomical structures through smaller skin incisions, minimal injury to the paraspinal muscles and ligamentous structures, and limited bone resection [4]. This tissue-preserving approach is associated with minimal blood loss, lower infection rates, reduced perineural fibrosis, minimized segmental instability, and lower levels of perioperative pain [5,6].

Despite these important advantages, endoscopic techniques are not completely risk-free. The literature reports an overall complication rate of 9.26% in endoscopic spine surgery, with most complications occurring during the perioperative period [7]. Stable management of irrigation fluid dynamics is essential for safe and effective procedures. Irrigation fluid dynamics are influenced by the portal diameter, height difference between the portals, flow resistance, and applied irrigation pressure [8]. Maintaining appropriate and balanced irrigation pressure is critical for establishing an adequate working space, controlling epidural bleeding, removing blood and surgical debris, and maintaining a clear surgical field [9,10].

Disruption of the balance between fluid inflow and outflow may lead to serious irrigation-related neurological and cardiovascular complications [11]. In addition to major complications such as incidental durotomy, cauda equina syndrome, transient neurological deficits, altered consciousness, seizures, visual disturbances, cardiac arrhythmias, and hypertensive episodes, minor complications including postoperative head and neck pain and paresthesia are among the most frequently reported irrigation-related complications [12]. These complications may have devastating consequences if not anticipated by the surgeon.

As the use of endoscopic spine surgery continues to increase, a better understanding of the technical aspects specific to this technique and its potential complications is becoming increasingly important. The available literature on fluid management and irrigation-related complications remains limited, and comparing different irrigation methods in terms of surgical efficacy, patient outcomes, and complications is critical for optimizing surgical safety. This study aimed to compare gravity-based and pump-assisted irrigation systems in patients undergoing UBE decompression surgery for LSS with respect to clinical and functional outcomes and to evaluate the effects of these two methods on operative time and complication rates.

## 2. Materials and Methods

### 2.1. Patient Selection

After obtaining approval from the Ethics Committee of Bandırma Onyedi Eylül University Faculty of Medicine (protocol code E-67961857-900-2600034002; approval date: 6 May 2026), the medical records of patients who underwent UBE decompression surgery between 1 June 2023 and 1 January 2026 were retrospectively reviewed.

Patients aged ≥50 years who underwent UBE decompression for clinically and radiologically confirmed single-level LSS that remained symptomatic despite appropriate conservative treatment were eligible for inclusion.

Patients with multilevel LSS, spondylolisthesis, non-degenerative spinal pathologies (vertebral fracture, tumor, or infection), previous lumbar spine surgery, incomplete clinical data, or incomplete follow-up were excluded from the study.

Two different irrigation systems, gravity-based and pump-assisted, were used in this study. Patients were not randomized; the irrigation method was determined according to the availability of the pump-assisted irrigation device at the time of surgery and was not based on medical or clinical considerations, including patient characteristics, disease severity, surgical indication, or surgeon preference. Patients were divided into two groups according to the irrigation system used: gravity-based and pump-assisted irrigation.

### 2.2. Clinical Evaluation

All patients were preoperatively evaluated using lumbar magnetic resonance imaging (MRI) and radiographs (anteroposterior, lateral, and flexion–extension). Radiological confirmation of single-level LSS was based on MRI evidence of central canal and/or lateral recess stenosis at a single lumbar level corresponding to the patient’s clinical symptoms. UBE decompression surgery was performed in patients with persistent symptoms despite appropriate conservative treatment. Patients were evaluated at postoperative week 1 and at 1, 3, and 6 months postoperatively. Follow-up evaluations after the sixth postoperative month were performed at 6-month intervals.

Pain severity was assessed preoperatively and at postoperative months 1 and 6 using the Numeric Rating Scale (NRS; 0 = no pain, 10 = worst imaginable pain) [13]. Functional disability was evaluated using the Oswestry Disability Index (ODI; score range, 0–100, with higher scores indicating greater disability) [14]. In addition, data on age, sex, follow-up time, surgical level, decompression type, operative time, bleeding control time, and complications were recorded.

Complications were additionally classified as major or minor according to the framework proposed by Glassman et al. [15], in which major complications were defined as events requiring additional surgical intervention or resulting in permanent or major neurological deficits, whereas general medical adverse events and perioperative events with transient clinical effects were classified as minor complications.

All surgical procedures were documented using video recordings. Operative time was defined as the time interval between the portal skin incision and the completion of the endoscopic procedure. The bleeding control time was calculated by a single investigator through retrospective review of the surgical video recordings as the total duration during which the radiofrequency ablation probe was actively used for intraoperative hemostasis. The investigator was blinded to the irrigation method used during each procedure.

### 2.3. Surgical Technique and Irrigation Management

A standard interlaminar UBE decompression technique was used in all patients [16]. All procedures were performed by a single surgeon who had completed the learning curve for unilateral biportal endoscopic spine surgery and had performed more than 100 UBE procedures before the study period. Under general anesthesia, the patients were placed in the prone position with support pads positioned beneath the thorax and pelvis to reduce intra-abdominal pressure. After confirming the target level under fluoroscopic guidance, cranial and caudal portals were created approximately 1 cm lateral to the midline in accordance with the standard UBE surgical technique [16]. The cranial portal was used as the viewing portal, and the caudal portal served as the working portal.

A 4-mm, 30° rigid arthroscope (REF 502-477-031; Stryker Endoscopy, Kalamazoo, MI, USA) was used for visualization. Irrigation fluid was introduced through the cranial portal and allowed to drain via the caudal portal. In accordance with the primary focus of this study, fluid flow within the surgical field was carefully controlled to maintain a balanced inflow–outflow relationship for both irrigation methods. The decompression technique was planned according to the location of the stenosis; unilateral decompression was performed for lateral recess stenosis, whereas bilateral decompression was performed for central stenosis. At the end of the procedure, a surgical drain was placed in each patient to reduce the risk of postoperative hematoma.

#### 2.3.1. Gravity-Based Irrigation System

**Figure 1:**
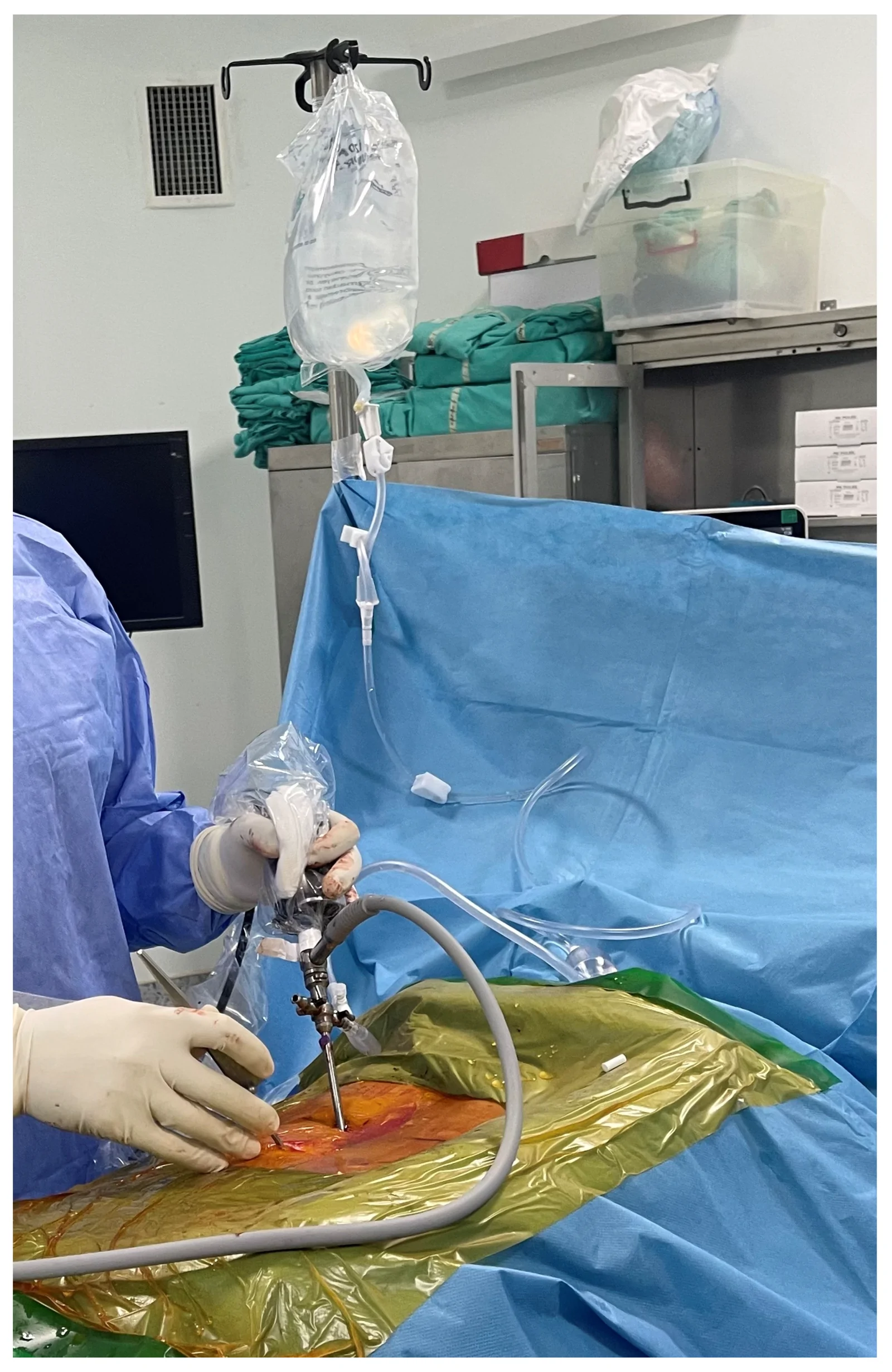
Gravity-based irrigation system used during UBE decompression surgery.

In the gravity-based irrigation system, a 3000-mL bag of isotonic sodium chloride solution was suspended approximately 50–60 cm above the surgical field, and the irrigation pressure was generated according to the height difference between the fluid source and working area (Figure 1). As previously described, this height corresponds to an irrigation pressure of approximately 30–40 mmHg [16]. The irrigation pressure was adjusted according to the surgical requirements by changing the height of the fluid bag.

#### 2.3.2. Pump-Assisted Irrigation System

**Figure 2:**
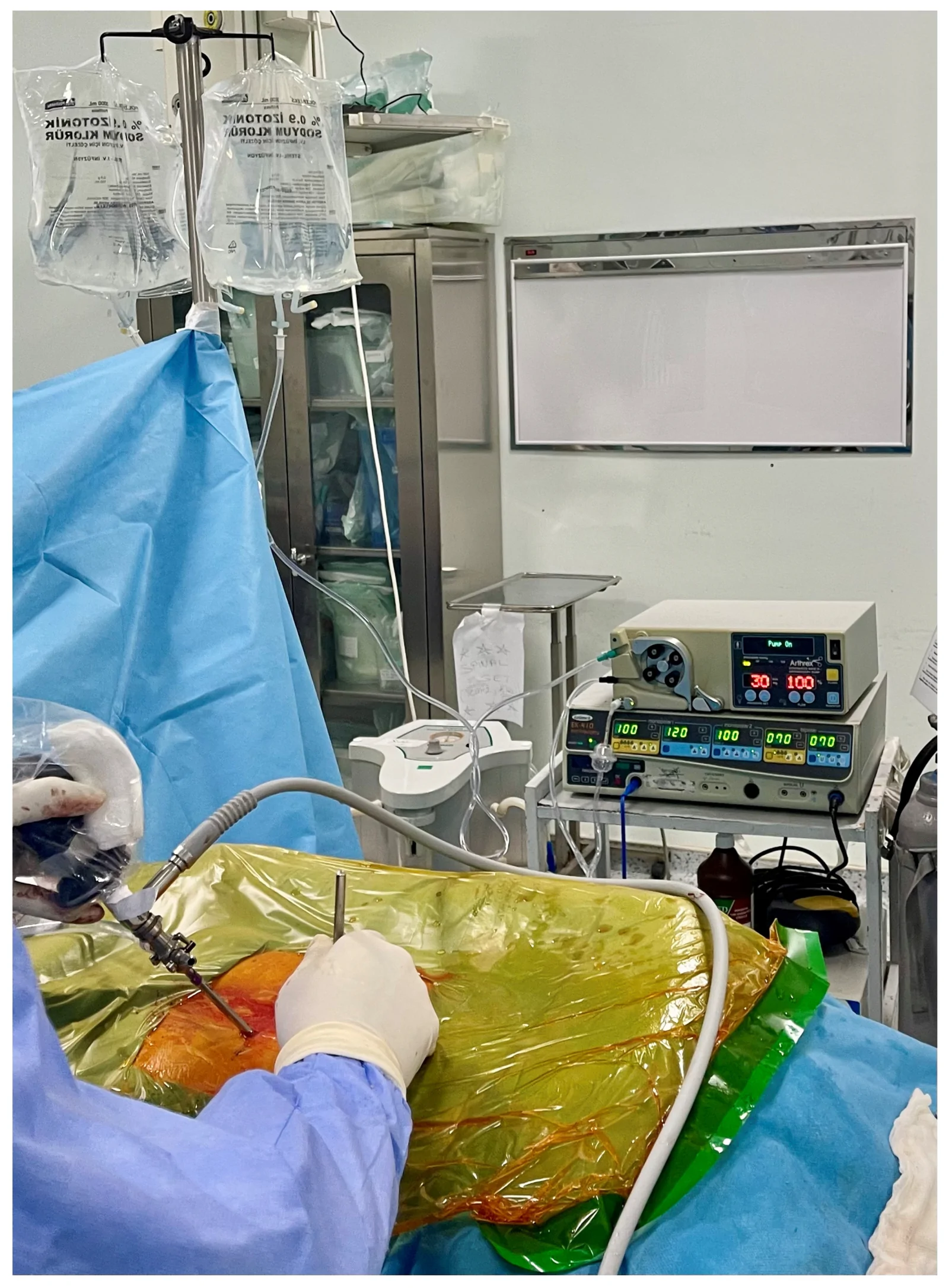
Pump-assisted irrigation system used UBE decompression surgery.

A pressure-controlled irrigation device (AR-6475; Arthrex Inc., Naples, FL, USA) was used in the pump-assisted irrigation system (Figure 2). The inflow pressure was maintained at 30–40 mmHg, within the safe range recommended in the literature [16], to provide a constant, non-pulsatile flow. Fluid outflow was achieved exclusively through the working portal (inflow-only system). When visualization was impaired due to bleeding, the irrigation pressure was temporarily increased; however, pressures exceeding 40 mmHg were not used.

### 2.4. Statistical Analysis

Statistical analyses of the study data were performed using IBM Statistical Package for the Social Sciences (SPSS) for MacOS version 30.0 (IBM Corp., Armonk, NY, USA). The distribution characteristics of continuous variables were evaluated using the Kolmogorov–Smirnov test, histogram inspection, and skewness and kurtosis values (±2). Categorical variables are presented as numbers and percentages (*n* [%]), whereas continuous variables are expressed as mean ± standard deviation.

Student’s *t*-test was used for comparisons between the groups. Pearson’s chi-square test or Fisher’s exact test was used for comparisons of categorical variables. Because baseline differences in age and sex were observed between the groups, changes in clinical scores over time according to the surgical technique were analyzed using repeated-measures analysis of covariance (ANCOVA), with time defined as the within-subject factor, surgical technique as the between-subject factor, and age and sex included as covariates to control for their potential confounding effects. The assumption of sphericity was assessed using Mauchly’s test. When the assumption of sphericity was violated, the Greenhouse–Geisser correction was applied.

Multivariable linear regression analysis was performed to identify the independent factors affecting operative time. Factors associated with the development of complications were evaluated using multivariable logistic regression analysis. Because baseline differences in age and sex were observed between the groups, these variables were included in the multivariable linear regression model to control for their potential confounding effects on the results. The multivariable logistic regression model used to evaluate the development of complications included irrigation type, decompression type, and operative time. In all statistical analyses, a two-tailed *p*-value of <0.05 was considered statistically significant.

### 2.5. Post Hoc Power Analysis

A post hoc power analysis was performed to evaluate the statistical adequacy of the study cohort for the primary outcome measures, namely operative time and complication rate. All calculations were based on a two-sided significance level of α = 0.05. For operative time, the statistical power was calculated as 94% using Cohen’s *d* effect size of 0.75. For the complication rate, the statistical power was calculated as 83% based on an absolute difference of 15% between the groups. Accordingly, the post hoc power analysis demonstrated that the study cohort (*n* = 107) provided sufficient statistical power for the primary outcome measures.

## 3. Results

The distribution of the demographic and surgical characteristics of the patients’ is shown in Table 1. A total of 107 patients were included in the study, of whom 51 (47.7%) received gravity-based irrigation and 56 (52.3%) received pump-assisted irrigation. Significant differences in sex (*p* = 0.014) and age (*p* = 0.021) distributions were observed between the groups (*p* = 0.014). No statistically significant differences were observed between the groups in terms of decompression type (unilateral/bilateral) or follow-up time (*p* > 0.05). Operative and bleeding control times were significantly shorter in the pump-assisted irrigation group (*p* < 0.001). No significant difference was found between the groups in terms of the distribution of surgical levels (*p* = 0.752).

**Figure 3:**
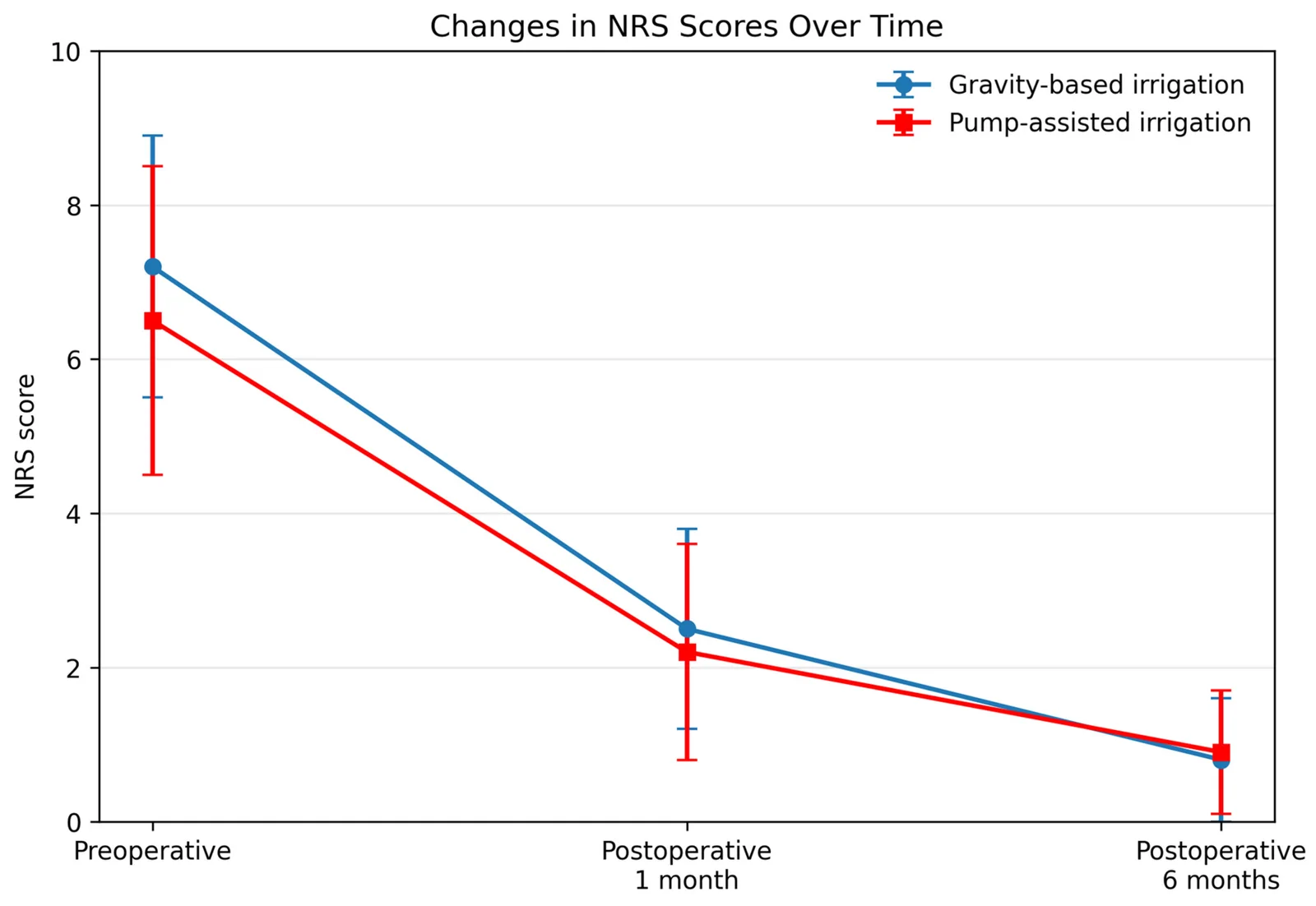
Changes in NRS scores over time in the gravity-based and pump-assisted irrigation groups. Values are presented as mean ± standard deviation.

**Figure 4:**
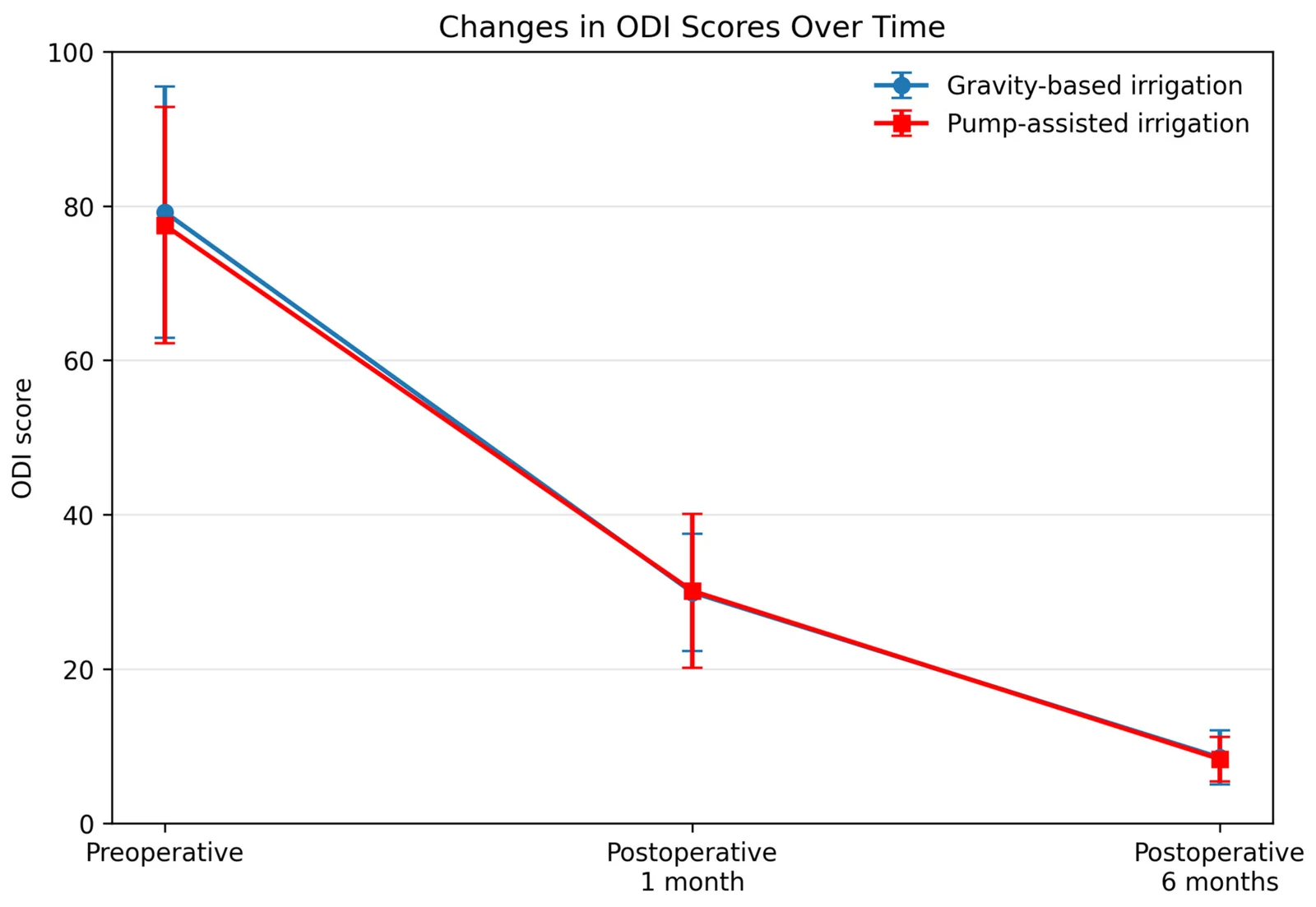
Changes in ODI scores over time in the gravity-based and pump-assisted irrigation groups. Values are presented as mean ± standard deviation.

Table 2 presents the time-dependent changes in the clinical scores according to the irrigation technique after adjustment for baseline differences in age and sex. Significant improvements in both NRS and ODI scores were observed over time (time effect, *p* < 0.001 for both). No significant differences were found between the groups in the NRS (group effect, *p* = 0.281) or ODI scores (group effect, *p* = 0.834). Likewise, no significant group-by-time interaction was observed for either the NRS (*p* = 0.362) or ODI (*p* = 0.647), indicating that the temporal patterns of improvement were comparable between the two irrigation techniques. Figure 3 and Figure 4 provide graphical illustrations of the changes in NRS and ODI scores over time, respectively, in both groups.

The results of the multivariable linear regression analysis performed to determine the factors affecting operative time are presented in Table 3. In the analysis, irrigation type, age, sex, and decompression type (unilateral/bilateral) were included in the model. As a result of the analysis, the pump-assisted irrigation system was found to have an independent effect on operative time, shortening it by an average of 8 min compared with the gravity-based irrigation method (*p* < 0.001). When the type of decompression was evaluated, the operative time was significantly longer in patients who underwent bilateral decompression than in those who underwent unilateral decompression. Bilateral decompression was an independent factor that increased operative time by an average of 24.5 min (*p* < 0.001). In contrast, age (*p* = 0.948) and sex (*p* = 0.528) had no statistically significant effects on operative time.

The distribution of complications according to the irrigation technique is presented in Table 4. Although the overall complication rate was lower in the pump-assisted irrigation group, this difference did not reach statistical significance (*p* = 0.052). Similarly, no statistically significant differences were observed between the groups in either major (*p* = 0.188) or minor (*p* = 0.345) complication rates. The results of the multivariable logistic regression analysis evaluating the factors associated with overall complication development are presented in Table 5. The analysis demonstrated that pump-assisted irrigation tended to reduce the risk of complications compared with gravity-based irrigation; however, this association did not reach statistical significance (OR = 0.408; *p* = 0.096). Likewise, neither operative time (OR = 1.007; *p* = 0.766) nor decompression type (OR = 1.258; *p* = 0.778) was identified as an independent predictor of complications.

## 4. Discussion

In this study, we compared gravity-based and pump-assisted irrigation systems in patients undergoing UBE decompression for LSS. The findings demonstrated that pump-assisted irrigation significantly reduced operative and bleeding control times, thereby improving surgical efficiency while providing clinical and functional outcomes comparable to those achieved with gravity-based irrigation. Although no statistically significant difference was observed between the groups regarding complication rates, a lower complication rate was observed in the pump-assisted irrigation group. Furthermore, multivariable analyses confirmed that pump-assisted irrigation was an independent predictor of shorter operative time. These findings indicate that pump-assisted irrigation may improve operative efficiency while maintaining comparable clinical outcomes.

One of the most important findings of this study was that the pump-assisted irrigation system significantly reduced both operative and bleeding control times compared with gravity-based irrigation. Multivariable linear regression analysis demonstrated that pump-assisted irrigation independently shortened the operative time by approximately 8 min. This finding is consistent with the controlled study by Guan and Wu [17], who compared pump-assisted and gravity-based irrigation during percutaneous endoscopic lumbar discectomy. In their study, the mean operative time was 65 min in the pump-assisted group and 74 min in the gravity-based group, with a statistically significant difference. They also reported that pump-assisted irrigation significantly reduced the intraoperative blood loss. This finding supports the shorter bleeding control time observed in the pump-assisted irrigation group in our study and suggests that pressure-controlled irrigation may provide more effective hemostasis than traditional gravity-based irrigation systems. Taken together, these findings suggest that pressure-controlled fluid management may contribute to a shorter operative time by providing more effective hemostasis and a more stable surgical field.

Guan and Wu [17] reported that pump-assisted irrigation provided more effective suppression of epidural venous bleeding and maintained a more stable surgical field through continuous, pressure-controlled fluid flow. Similarly, continuous irrigation improves visualization during biportal endoscopic surgery [18]. These mechanisms may substantially improve procedural efficiency in UBE surgery, where the working space is limited and visualization largely depends on irrigation dynamics. Previous studies have emphasized that a stable and clear surgical field is a key determinant of operative efficiency in endoscopic spine surgery [8,17,18,19]. In our study, the shorter bleeding control and operative times observed in the pump-assisted irrigation group also support this finding.

In our study, operative time was significantly longer in patients who underwent bilateral decompression than in those who underwent unilateral decompression. This finding is in direct agreement with the procedure-specific comparison reported by Xu et al. in their UBE surgical series [20]. Xu et al. compared bilateral decompression and unilateral discectomy procedures within the same retrospective cohort with respect to operative time and reported that bilateral decompression significantly increased operative time. In the cumulative analysis of the same study, the bilateral procedure consistently required a longer operative time than the unilateral procedure across all phases of the learning curve. Therefore, the difference in operative time was a procedure-specific characteristic independent of the surgeon’s experience level. In this context, the longer operative time observed for bilateral decompression in our study may be explained by the need for contralateral sublaminar bone work and removal of the ligamentum flavum.

In our study, the overall complication rate was lower in the pump-assisted irrigation group, and this difference was close to being statistically significant. Similarly, no statistically significant differences were observed between the groups in either major or minor complication rates. Furthermore, multivariable logistic regression analysis demonstrated a strong trend toward a reduced risk of complications with pump-assisted irrigation; however, this association was not statistically significant. Similarly, Guan and Wu [17] demonstrated a significantly lower incidence of complications in patients treated with pump-assisted irrigation than in those treated with gravity-based irrigation. In their study, no symptoms of increased intracranial pressure were observed in the pump-assisted irrigation group, whereas lower rates of nerve root injury and postoperative recurrence were reported in the same group. These findings are clinically important and suggest that the irrigation method may influence surgical safety.

In endoscopic spine surgery, irrigation is an important technical component that can directly affect the epidural and intradural pressure dynamics. Previous studies have shown that inadequate fluid outflow, excessive irrigation pressure, or disruption of dural integrity may increase epidural and intracranial pressure, thereby predisposing patients to various neurological complications [11,12]. In contrast, Wang et al. [21] demonstrated that insufficient irrigation pressure may adversely affect surgical outcomes by increasing perioperative blood loss. Therefore, maintaining the irrigation pressure within an optimal range is critical for the safe performance of endoscopic spine surgery.

The lower trend in complications observed in the pump-assisted irrigation group in our study may be explained by the principles of the fluid dynamics. In gravity-based systems, the irrigation pressure is determined by the height of the fluid bag and cannot adapt to intraoperative changes in flow resistance [22]. In contrast, pump-assisted systems can regulate irrigation flow according to changes in resistance within the surgical field, thereby maintaining a more stable pressure [17,22]. Yu et al. [22] measured working-space pressure in real time and reported that pump-assisted irrigation systems provided significantly lower working-space pressure than gravity-based systems. Therefore, the lower and more stable working space pressure achieved with pressure-controlled fluid management may be considered an important advantage that could reduce the risk of complications.

Nevertheless, according to the multivariable logistic regression analysis, neither the type of decompression nor the operative time was associated with the development of complications. In contrast, Vargas et al. [11] suggested that irrigation duration may be a potential risk factor for the development of complications. Consistent with this view, a case of generalized tonic–clonic seizure following prolonged irrigation and the use of a large volume of normal saline after percutaneous endoscopic lumbar discectomy has been reported, emphasizing the need for careful irrigation management [23]. However, technical factors, such as surgical experience, learning curve, and instrumentation used, may also influence outcomes [24]. Although the role of surgical experience in the development of irrigation-related complications has not been fully elucidated, these complications are more likely to occur in technically demanding procedures with prolonged operative times [11]. In our study, all procedures were performed by an experienced surgeon, the mean operative time was relatively short, and patients with complex multilevel LSS were excluded from the study. Therefore, no significant association was found between operative time, decompression type, and the development of complications.

In our study, significant improvements in the NRS and ODI scores were observed over time in both groups. After adjustment for baseline differences in age and sex, no significant differences were observed between the groups with respect to pain and functional outcomes. These findings indicate that both irrigation methods may provide comparable clinical success when adequate decompression is achieved. Our findings are consistent with comparative studies and meta-analyses reporting that UBE surgery provides clinical outcomes comparable to those of conventional surgical techniques [25,26,27]. Furthermore, the absence of a significant group-by-time interaction for the NRS and ODI scores indicates that the postoperative recovery trajectory was similar between the two irrigation techniques.

This study had several important strengths. To the best of our knowledge, no previous clinical study has comprehensively compared gravity-based and pump-assisted irrigation systems in UBE decompression surgery for LSS with respect to operative efficiency, clinical and functional outcomes, and irrigation-related complications. Given that many perioperative complications reported during UBE surgery are related to irrigation management and that an optimized irrigation strategy has not yet been established, this study addresses an important gap in the literature. Another important strength of this study was the inclusion of a homogeneous study population. This approach reduced the influence of potential confounding factors and allowed a more reliable evaluation of the effects of irrigation methods on surgical and clinical outcomes. Furthermore, all surgical procedures were performed by a single surgeon experienced in endoscopic spine surgery using a standardized surgical technique, thereby minimizing variability related to the surgical technique. In addition, operative time and bleeding control time were objectively determined through retrospective review of intraoperative video recordings by a single blinded investigator, thereby reducing the potential for observer bias. The use of multivariable regression analysis to control for potential confounding factors further strengthens the methodological robustness of our findings.

This study had several limitations. First, its retrospective design and the lack of randomization represent inherent methodological limitations that may have introduced residual selection bias. Although treatment allocation was based on the availability of the pump-assisted irrigation device rather than patient characteristics or surgeon preference, residual confounding cannot be completely excluded. Furthermore, the operating surgeon had completed the learning curve for UBE surgery before the study period; however, the potential influence of temporal changes and the continued accumulation of surgical experience during the study period cannot be completely excluded. Second, although bleeding control time was assessed by a single blinded investigator, intraobserver reproducibility and formal reliability analyses were not performed. Third, the study was conducted at a single center, which may limit the generalizability of our findings.

Given these limitations, future studies should focus on prospective, multicenter, randomized designs to validate the present findings and further determine the optimal irrigation strategy for UBE decompression surgery in terms of operative efficiency, clinical outcomes, and surgical safety.

## 5. Conclusions

Pump-assisted irrigation significantly reduced operative and bleeding control times compared with gravity-based irrigation while providing comparable clinical and functional outcomes in patients undergoing UBE decompression for LSS. Although the reduction in complication rates did not reach statistical significance, pump-assisted irrigation showed a consistent trend toward improved surgical safety. These findings indicate that pump-assisted irrigation may represent an effective irrigation strategy that improves operative efficiency and may contribute to surgical safety in UBE decompression surgery.

## Figures and Tables

**Table 1:** Demographic and Surgical Characteristics.

Variables	Gravity-Based Irrigation 51 (47.7)	Pump-Assisted Irrigation 56 (52.3)	Total	*p*-Value
Sex				0.014
Male	17 (33.3)	32 (57.1)	49 (45.8)	
Female	34 (66.7)	24 (42.9)	58 (54.2)	
Age	69 ± 6	72 ± 5	70 ± 6	0.021
Decompression type				0.086
Unilateral	18 (35.3)	29 (51.8)	47 (43.9)	
Bilateral	33 (64.7)	27 (48.2)	60 (56.1)	
Follow-up (months)	15.4 ± 7.2	14.2 ± 6.9	14.8 ± 7.1	0.378
Operative time (min)	76.4 ± 17.1	64.1 ± 14.3	69.9 ± 16.8	<0.001
Bleeding control time (min)	6 ± 1.4	4.4 ± 1.2	5.1 ± 1.5	<0.001
Surgical level				0.752
L2–3	2 (3.9)	3 (5.4)	5 (4.7)	
L3–4	18 (35.3)	15 (26.8)	33 (30.8)	
L4–5	22 (43.1)	29 (51.8)	51 (47.7)	
L5–S1	9 (17.6)	9 (16.1)	18 (16.8)	

Categorical variables are presented as *n* (%), and continuous variables are presented as mean ± standard deviation.

**Table 2:** Time-Dependent Changes in Clinical Scores.

Variables (*N* = 107)	Gravity-Based Irrigation (*n* = 51)	Pump-Assisted Irrigation (*n* = 56)	*p*-Value (Time)	*p*-Value (Group)	*p*-Value (Group × Time)
**NRS**			<0.001	0.281	0.362
Preoperative	7.2 ± 1.7	6.5 ± 2			
Postoperative 1st month	2.5 ± 1.3	2.2 ± 1.4			
Postoperative 6th month	0.8 ± 0.8	0.9 ± 0.8			
**ODI**			<0.001	0.834	0.647
Preoperative	79.2 ± 16.3	77.5 ± 15.3			
Postoperative 1st month	29.9 ± 7.6	30.1 ± 10			
Postoperative 6th month	8.5 ± 3.5	8.3 ± 2.9			

Changes in clinical scores over time according to the irrigation technique were analyzed using repeated-measures analysis of covariance (ANCOVA), with time defined as the within-subject factor, irrigation technique as the between-subject factor, and age and sex included as covariates to control for their potential confounding effects. Values are presented as mean ± standard deviation.

**Table 3:** Results of Multivariable Linear Regression Analysis of Factors Affecting Operative Time.

Variables	B	Standard Error	t	*p*-Value
Irrigation type	−8.000	2.067	−3.871	<0.001
Age	0.011	0.175	0.065	0.948
Sex	1.288	2.035	0.633	0.528
Decompression type	24.515	2.012	12.185	<0.001

Model Summary: R = 0.812; R^2^ = 0.659; Adjusted R^2^ = 0.645; F = 49.240; *p* < 0.001. Irrigation type: 1 = Gravity-based, 2 = Pump-assisted; Sex: 1 = Male, 2 = Female; Decompression: 1 = Unilateral, 2 = Bilateral.

**Table 4:** Complications.

	Gravity-Based Irrigation	Pump-Assisted Irrigation	Total	*p*-Value
**Complications**	14 (27.5)	7 (12.5)	21 (19.6)	0.052
**Major complications**	7 (13.7)	3 (5.4)	10 (9.3)	0.188
Dural damage	3 (5.9)	1 (1.8)	4 (3.7)	
Foot drop	1 (2)	0 (0)	1 (0.9)	
Transient weakness	2 (3.9)	1 (1.8)	3 (2.8)	
Transient amnesia	1 (2)	0 (0)	1 (0.9)	
Seizures	0 (0)	1 (1.8)	1 (0.9)	
**Minor complications**	7 (13.7)	4 (7.1)	11 (10.3)	0.345
Head and neck pain	3 (5.9)	1 (1.8)	4 (3.7)	
Paresthesia	4 (7.8)	2 (3.6)	6 (5.6)	
Tinnitus	0 (0)	1 (1.8)	1 (0.9)	

Categorical variables are presented as *n* (%).

**Table 5:** Results of Multivariable Logistic Regression Analysis of Factors Associated with Complications.

Variables	OR (95 CI)	*p*-Value
Irrigation type		
Gravity-based	Reference	-
Pump-assisted	0.408 (0.142–1.172)	0.096
Operative time (min)	1.007 (0.960–1.057)	0.766
Decompression type		
Unilateral	Reference	-
Bilateral	1.258 (0.255–6.208)	0.778

OR: Odds ratio; CI: Confidence interval.

## Data Availability

The data supporting the findings of this study are available upon request from the corresponding author. The data are not publicly available because of privacy or ethical restrictions.

## Abbreviations

LSS	Lumbar spinal stenosis
UBE	unilateral biportal endoscopic
MRI	magnetic resonance imaging
NRS	Numeric Rating Scale
ODI	Oswestry Disability Index
SPSS	Statistical Package for the Social Sciences
OR	Odds ratio
CI	Confidence interval
